# Spatial Analysis of Wildlife Tuberculosis Based on a Serologic Survey Using Dried Blood Spots, Portugal

**DOI:** 10.3201/eid2412.171357

**Published:** 2018-12

**Authors:** Nuno Santos, Telmo Nunes, Carlos Fonseca, Madalena Vieira-Pinto, Virgílio Almeida, Christian Gortázar, Margarida Correia-Neves

**Affiliations:** University of Minho School of Medicine (ICVS), Braga, Portugal (N. Santos, M. Correia-Neves);; Portugal Government Associate Laboratory, Braga/Guimarães, ICVS/3B’S, Portugal (N. Santos, M. Correia-Neves);; University of Lisbon, Lisbon, Portugal (T. Nunes, V. Almeida);; University of Aveiro, Aveiro, Portugal (C. Fonseca);; University of Trás-os-Montes e Alto Douro, Vila Real, Portugal (M. Vieira-Pinto);; Instituto de Investigación en Recursos Cinegéticos, Ciudad Real, Spain (C. Gortázar)

**Keywords:** wildlife tuberculosis, bovine tuberculosis, Mycobacterium bovis, bacteria, tuberculosis and other mycobacteria, respiratory infections, epidemiologic surveillance, serologic survey, dried blood spots, spatial epidemiology, cattle, wild boar, red deer, wild animals, zoonoses, Portugal

## Abstract

We investigated the spatial epidemiology of bovine tuberculosis (TB) in wildlife in a multihost system. We surveyed bovine TB in Portugal by serologic analysis of elutes of dried blood spots obtained from hunted wild boar. We modeled spatial disease risk by using areal generalized linear mixed models with conditional autoregressive priors. Antibodies against *Mycobaterium bovis* were detected in 2.4% (95% CI 1.5%–3.8%) of 678 wild boar in 2 geographic clusters, and the predicted risk fits well with independent reports of *M. bovis* culture. Results show that elutes are an almost perfect substitute for serum (Cohen unweighted κ = 0.818), indicating that serologic tests coupled with dried blood spots are an effective strategy for large-scale bovine TB surveys, using wild boar as sentinel species. Results also show that bovine TB is an emerging wildlife disease and stress the need to prevent further geographic spread and prevalence increase.

Bovine tuberculosis (TB) is a zoonotic disease caused by *Mycobacterium bovis* and other members of the *M. tuberculosis* complex, whose natural hosts are wild and domestic mammals (*1*). Bovine TB is a disease of economic and public health relevance and is subjected to mandatory control programs in livestock in many countries. As a result of these programs, bovine TB has been eradicated in regions such as Australia and Scandinavia. However, in other regions, persistence of infection has been attributed to wildlife reservoirs, such as cervids in North America (*2*). In the Iberian Peninsula (Figure 1, panel A), bovine TB is maintained in a multihost pathogen system in which *M. bovis* and *M. caprae* circulate between sympatric wild ungulates (mainly wild boar [*Sus scrofa*] and red deer [*Cervus elaphus*]) and free-ranging domestic ungulates (*1*).

**Figure 1:**
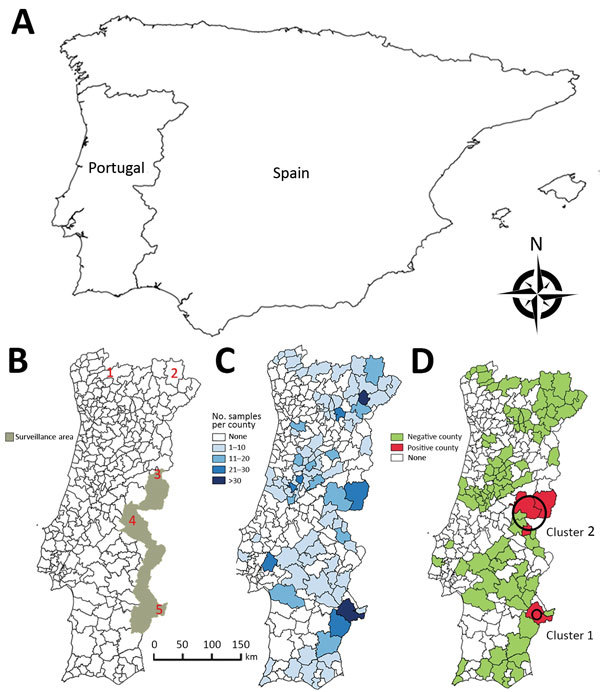
Choropleth maps for spatial study of bovine tuberculosis (TB) in wildlife, Portugal. A) Iberian Peninsula. B) Official surveillance area for bovine TB in large game species. Red numbers indicate historical population refuges of wild ungulates: 1) Gerês, 2) Montesinho, 3) Malcata, 4) São Mamede, and 5) left bank of the Guadiana River. C) Distribution of serologic samples analyzed per county. D) Distribution of bovine TB–positive samples. Black circles indicate the 2 clusters identified.

In Portugal, control of bovine TB has resulted in a low prevalence in livestock (4.5 cases/10,000 cattle and 2.9 cases/1,000 herds) in 2017 (*3*). Nevertheless, disease incidence has stabilized in recent years, and awareness of wildlife hosts has fueled the discussion over their role as reservoirs of bovine TB. In 2011, the Portuguese Animal Health Directorate (Lisbon, Portugal) established a surveillance area for bovine TB in large game species, encompassing regions where the disease was known to be present in wild ungulate populations (Figure 1, panel B).

Bovine TB in wildlife shows spatial structuring in the Iberian Peninsula. There is a core area in the central–southwestern region, in which the average prevalence of macroscopic lesions is 59% in wild boar (*4*). At the periphery of this core area, prevalence decreases, and becomes low to undetectable in eastern, northern, and western regions of the Iberian Peninsula (*5*–*7*). Nevertheless, spatial analyses of bovine TB on wildlife in the Iberian Peninsula, other than disease mapping, are notably lacking.

Large-scale disease surveys in wildlife require mass-scalable and inexpensive diagnostic tests; serologic methods are one of the most suitable techniques (*8*). An ELISA for detecting antibodies against the *M. tuberculosis* complex has been described and validated for use in wild boar (*9*,*10*) and showed a moderately high estimated sensitivity of 79.2% and an excellent specificity of 100% (*10*). Another improvement for large-scale disease surveys in wildlife is a sampling protocol that might be used by nonspecialized personnel, such as hunters. One example is the dried blood spot technique, which was originally developed for human sampling but has been increasingly used for wildlife disease surveys (*11*).

Wild boar have been shown to be a maintenance host for bovine TB in the Iberian Peninsula (*12*). Furthermore, wild boar have been used as a sentinel for bovine TB in wildlife because of their high susceptibility to infection with *M. bovis* and *M. caprae* and extensive exposure to these pathogens through direct contact, necrophagic habits, or fossorial habits (*13*–*15*). Wild boar populations in Portugal reached their nadir in the middle of the 20th century, when the species survived in only 5 populations or historical refuges (*16*,*17*) (Figure 1, panel B). Since that time, wild boar populations have increased markedly and are currently found throughout Portugal (*16*,*17*).

To assess the spatial epidemiology of bovine TB in wildlife, we analyzed the multihost pathogen system in the Iberian Peninsula by using the wild boar as a sentinel species. The main aims of this study were to assess the performance of dried blood spots as an alternative sample collection and storage technique for serologic surveys of bovine TB, map the distribution of wildlife bovine in Portugal on the basis of serologic methods, investigate spatial clustering of bovine TB in wildlife, and model the risk for bovine TB in Portugal.

## Methods

### Collection of Samples

Biological samples were obtained from 678 wild boar hunted during 2006–2013. Of these samples, 107 were serum and 571 were dried blood spots collected on Protein Saver (PS) 903 cards (Whatman, Maidstone, UK) (n = 308) or Flinders Technology Associates (FTA) paper (Whatman) (n = 263).

Serum samples were obtained from blood collected from the thoracic or abdominal cavities of hunted wild boar and stored at −20°C. Absorbent papers were distributed to hunters with instructions for the papers to be soaked in blood available from the thoracic cavity. Papers were dried in the shade and kept at room temperature in zipper-lock bags with data on the location of collection. Dried blood spots were recovered at the end of the hunting season and kept at −20°C until processed. Elutes were obtained by cutting half a circle of PS card or a quarter of FTA cards. According to the manufacturer’s instructions, a PS card absorbs 80 µL of blood, and an FTA card absorbs 125 µL of blood. These samples were further divided into 5 portions that were incubated overnight refrigerated in 200 µL of phosphate-buffered saline (PBS), and the elute obtained was subjected to serologic analysis. Paired samples of serum and dried blood spots were collected from 22 wild boar that had macroscopic lesions compatible with bovine TB.

In 1 region of southeastern Portugal, tissue samples were collected from 340 hunted wild boar with either bovine TB-compatible lesions or pooled lymph nodes when lesions were absent. These samples were obtained during 2009–2014 and kept at −20°C until bacteriological cultures were performed.

### Laboratory Analysis

We tested serum samples by using an ELISA and bovine purified protein derivative (bovine PPD) as antigen and protein G–horseradish peroxidase as conjugate (*10*). In brief, we coated wells of ELISA plates with 100 µg of bovine PPD for 18 hours at room temperature, washed the plates with PBS containing 0.05% Tween 20 (PBST), and incubated the plates for 1 hour at 37°C with 140 µL/well of 5% skimmed milk in PBST to block potential free binding sites. We added serum or elute samples to plates (10 µL/well) at dilutions of 1:200 in PBS for serum and 1:50 in PBS for elutes and incubated samples for 1 hour at 37°C. Protein G–horseradish peroxidase conjugate was added (100 µL/well) at a dilution of 2.5 µg/mL in PBST and incubated at 37°C for 80 min. A total of 100 µL of substrate (SigmaFast OPD; Sigma-Aldrich, St. Louis, MO, USA) was added to each well and incubated at room temperature in the dark. The reaction was stopped after 20 min by the addition of 50 µL/well of 3N H_2_SO_4_. 

We measured optical density (OD) by using a spectrophotometer at 450 nm. Blanks and positive and negative controls were tested in duplicate in each plate, and samples were tested in triplicate. Results were calculated as mean sample OD divided by 2 times the mean negative control OD; the cutoff for positivity was 1 (*10*).

We prepared bacteriological cultures of tissue samples in a Biosafety Level 3 facility according to a described protocol (*18*). In brief, 3 g of tissue were homogenized and decontaminated for 2 hours with 0.75% hexa-decylpyridinium chloride, centrifuged at 2,566 × *g* for 30 min, and the supernatant collected. We inoculated 2 tubes containing Coletsos medium (bioMerieux, Marcy l’Étoile, France) with 250 µL of supernatant–sediment interface and incubated these tubes at 37°C for 15 wks. Isolates were identified by PCR for 16S rRNA, insertion sequence 1561, and Rv1510 genes after DNA extraction by using the standard phenol-chloroform method after bead-beating with 100 μL of 0.1-mm zirconia/silica beads (Biospec Products, Bartlesville, OK, USA) in a FastPrep 24 Homogenizer (MP Biomedicals, Santa Ana, CA, USA).

### Data Analysis

To determine agreement between ELISA results (positive or negative) from paired serum and elute samples, we computed the Cohen unweighted κ value (*19*) by using irr in R software (*20*) (R Development Core Team, Vienna, Austria). We obtained serologic data for 92 of 278 counties in Portugal (Figure 1, panel C). The area considered for each county excluded regions classified as urban or water bodies in the CORINE database (*21*). We created choropleth maps, which use differences in shading, coloring, or placing of symbols within predefined areas to indicate average values of a property or quantity in those areas, of regions with bovine TB and performed spatial interpolation by using QGIS version 2.6.1 Brighton software (https://www.qgis.org/en/site/getinvolved/governance/governance.html). To detect spatial aggregation of bovine TB, we performed cluster analysis on the basis of Kuldorff spatial scan statistics by using the Bernoulli distribution and setting the maximum cluster size at 50% with SatScan version 9.3.1 software (*22*).

We assessed the association between detection of bovine TB in wild boar in each county and independent variables (Table 1) by using spatial generalized linear mixed modeling of areal data with the localized conditional autoregressive priors proposed by Lee and Mitchel (*23*). We included the bioregions of the Iberian Peninsula (Atlantic/Mediterranean) (*24*) as local conditional autoregressive priors because they have been shown to have distinct bovine TB epidemiologic scenarios (*7*). We implemented models by using the CARBayes version 5.0 package (*25*) in R software. We based inference on 20,000 Markov Chain Monte Carlo iterations (200,000 iterations with a thin factor of 10 to reduce autocorrelation) after an initial burn-in of 40,000 iterations. Taking into consideration the home ranges of wild boar (*26*), we decided to include counties <25 km apart in the neighborhood matrix of each other.

**Table 1:** Independent variables included in initial models of bovine tuberculosis, Portugal*

Variable	Specific variable	Unit
Wild host density	Wild boar hunting bag	Wild boar hunted/km^2^†
	Red deer hunting bag	Red deer hunted/km^2^†
	Fallow deer hunting bag	Fallow deer hunted/km^2^†
Game management	Intensity of management as proportion of area as tourist or national hunting zones	Proportion
Domestic host density	Cattle density >6 mo of age, meat	Cattle/km^2^
	Sheep density >6 mo of age, extensive	Sheep/km^2^
	Goat density >6 -mo of age, extensive	Goats/km^2^
	Pig density >6 mo of age, free range	Pigs/km^2^
Bovine tuberculosis incidence in cattle	Bovine tuberculosis incidence in cattle	Proportion
Historical population dynamics	Distance to the nearest historical refuge	km

*Spatial unit for all variables in county. †Average no. of each wild game species hunted per county per year/area of hunting areas for which data were available per county per year.

We estimated domestic host absolute densities on the basis of data from the Portuguese Livestock Movement Database (https://ifap.pt) with the following inclusion criteria: we considered only animals >6 months of age among free-range pigs, extensively reared sheep and goats, and meat production cattle. We excluded intensively reared animals to provide more realistic estimates of the livestock population at potential risk from contact with wildlife. Meat-production cattle were selected as a proxy for extensive rearing because this is the predominant beef cattle production system in Portugal, and dairy cattle herds are almost exclusively housed indoors.

We selected hunting bag (number of animals hunted per square kilometer in 1 year) as a proxy for wild ungulate density and calculated this density as the annual average of the number of hunted ungulates (wild boar, red deer, and fallow deer) from those hunting areas for which >2 years of data were available for 2008–2012. Because hunting bag data were not available for 65/278 counties, we performed an inverse distance weighted interpolation with power set at 3 to obtain estimates for the entire territory.

We selected the proportion of the area of each county dedicated to commercial hunting (tourism and national hunting areas usually intensively managed for maximizing profit) as a proxy for intensity of game management. Other types of hunting areas are dedicated to recreational hunting (i.e., usually no fencing, restocking, or large-scale artificial feeding of large game species). We included the historical presence of wild boar as the distance from the centroid of each county to the nearest historical refuge (Figure 1, panel B). We calculated the incidence of bovine TB in cattle as the average of the annual incidence rate for each county during 2008–2012 on the basis of data from the bovine TB eradication program.

We estimated a variance inflation factor to assess multicollinearity with a threshold of 5. We used a nonspatial logistic regression with backward stepwise elimination and selected the final model on the basis of Akaike information criterion corrected for small samples and model weights (*27*). We calculated the McFadden pseudo-R^2^ value to measure the goodness of fit of each model. We used the posterior probabilities of the spatial model to predict the risk for bovine TB in wild boar populations in all counties in Portugal. We assessed model convergence on the basis of the Geweke statistic (*28*).

## Results

We found that 11 paired serum samples and PS elutes were positive for antibodies against bovine PPD, 9 were negative for both, and 2 were positive in serum and negative in elutes. There was an almost perfect agreement between serologic results for both types of samples (mean ± SD Cohen unweighted κ value = 0.818 ± 0.121).

We detected antibodies against bovine PPD in 16/678 wild boar (2.4%, 95% CI 1.5%–3.8%). Antibody-positive wild boar originated from 4/92 counties tested (Figure 1, panel C). Kulldorff spatial scan statistics identified 2 clusters of bovine TB in wildlife. Cluster 1 was found in 1 county that had 8/36 positive wild boar (relative risk = 17.83, p<0.001), and cluster 2 was found in 5 counties (radius = 43 km) that had 8/65 positive wild boar (relative risk = 9.43, p = 0.011) (Figure 1, panel D).

**Figure 2:**
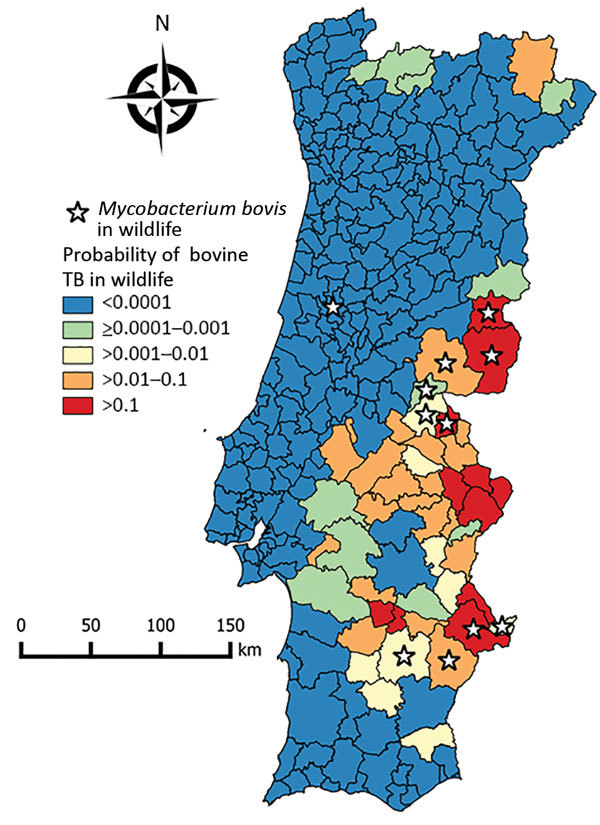
Choropleth map of risk for bovine TB in wildlife, Portugal, showing the probability of the presence of bovine TB in wildlife in counties based on the conditional autoregressive spatial generalized linear mixed model. Stars indicate counties in which *Mycobacterium bovis* was isolated from free-ranging wildlife, determined on the basis of independent published data (*6*,*29*–*32*). TB, tuberculosis.

The selected nonspatial logistic regression model with the presence of bovine TB as the dependent variable showed a McFadden pseudo-R^2^ value = 0.656 (Table 2). The spatial generalized linear mixed model (deviance information criterion = 24.141) that included the variables distance to historical refuge, bovine TB incidence in cattle, red deer hunting bag, intensity of management, and red deer hunting bag times intensity of management (Table 2) explained 45.5% of the deviance. The posterior probability of the presence of bovine TB in wildlife (Figure 2) was significantly higher for those counties with independent reports of isolation of *M. bovis* from free-ranging wildlife (Mann-Whitney–Wilcoxon W = 320; p<0.001) (*26**,**29**–**32*).

**Table 2:** Variables included in nonspatial binomial general linear and spatial generalized linear mixed models of bovine tuberculosis in wild boar, Portugal*

Variable	Logistic regression model		Spatial generalized linear mixed model	
			Median coefficient (95% credible interval)	Geweke statistic

Coefficient (95% CI)	p value
Intercept	−8.561 (−25.992 to −2.644)	0.073		−12.886 (−19.767 to −7.985)	1.3
Wild boar historical refuge	−0.114 (−0.356 to −0.020)	0.101		−0.088 (−0.170 to −0.022)	1.4
Bovine tuberculosis incidence in cattle	73.719 (6.919 to 231.075)	0.126		35.797 (−4.396 to 82.742)	−1.6
Management intensity	29.296 (6.167 to 101.249)	0.126		25.009 (6.510 to 49.979)	−1.3
Red deer hunting bag	9.391 (1.884 to 34.953)	0.150		6.990 (1.823 to 11.814)	−0.8
Red deer hunting bag × management intensity	−30.922 (−118.421 to −7.887)	0.162		−23.221 (−39.083 to −8.343)	0.9

**Figure 3:**
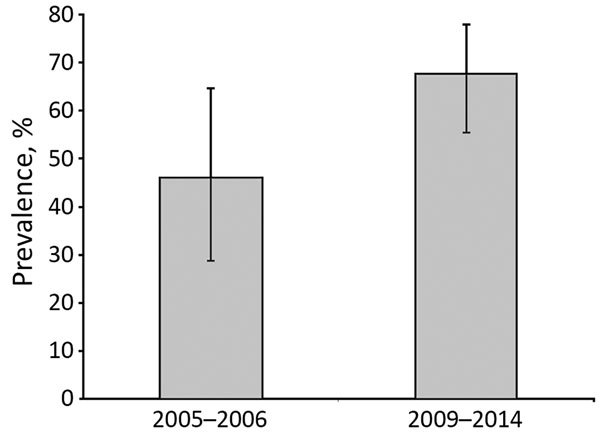
Temporal trend in prevalence of bovine tuberculosis in wild boar in 1 nonfenced hunting area, Portugal, by bacteriological culture during 2005–2006 (*6*) and 2009–2014 (this study). Error bars indicate 95% CIs.

We further investigated bovine TB in southeastern Portugal. This investigation included cluster 1, where *M. bovis* (n = 51) and *M. caprae* (n = 2) were isolated from 53/340 wild boar in 6/17 hunting areas, which had an overall bacteriological culture prevalence of 15.6% (95% CI 12.1%–19.8%). In 1 nonfenced hunting area, culture prevalence of wild boar bovine TB increased significantly (p = 0.049 by Fisher exact test) from 46.2% (95% CI 26.6%–66.6%) during 2005–2006 (*6*) to 67.7% (95% CI 55.4%–78.0%) during 2009–2014 (Figure 3).

## Discussion

We report a spatial analysis of bovine TB in wildlife in the multihost system of the Iberian Peninsula on the basis of serologic data for hunted wild boar. Serologic analysis was previously shown to have reasonably good sensitivity and excellent specificity for detecting bovine TB in wild boar (*10*), and this species is a suitable sentinel for bovine TB (*13*–*15*). We provide evidence showing that dried blood spots collected on PS cards by nonspecialist personnel are appropriate for serologic surveys of bovine TB, as shown by the almost perfect agreement between serologic results for serum samples and elutes. These results were expected because dried blood spots have been extensively validated for many other human or animal host–pathogen systems and generally found to be suitable for serologic surveys, even when blood is collected in remote and demanding conditions (*11*,*33*). This combination of dried blood spots and serologic analysis is a major advance for large-scale surveillance of bovine TB in wildlife anywhere wild boar are a suitable indicator species.

Our results support previous data suggesting a strong spatial structure of bovine TB in wildlife. Two clusters were identified in southern (cluster 1) and central-eastern (cluster 2) Portugal, which are located at the periphery of the high-prevalence core area in the central-southwestern region of the Iberian Peninsula (*4*,*5*). A subsequent survey for bovine TB in cluster 1 relied on bacteriological culture of tissues collected from hunted wild boar, which is an established but more expensive and labor-intensive diagnostic technique than serologic analysis. This regional bacteriological survey confirmed bovine TB as an emerging disease in wildlife and documented a 46.2% increase in prevalence in less than a decade at 1 nonfenced hunting area, similar to other populations in the Iberian Peninsula (*4*).

The spatial risk model we reported identified some predicted high-risk counties not included in the surveillance area for bovine TB in large game species (Figure 1, panel B) and thus could be used for better allocation of resources for wildlife disease surveillance and public health protection. The spatial model of bovine TB risk in wildlife generally agrees with results of published independent surveys because most reported wildlife isolates of *M. bovis* and *M. caprae* overlap with predicted moderate-risk to high-risk areas. The single exception is *M. bovis* isolated from a wild boar in Coimbra in central-western Portugal (Figure 2), an area that has a low predicted risk for bovine TB in wildlife (*30*). Red deer were introduced into this region during 1995–1999 and some of the founder animals originated from bovine TB–infected areas included in cluster 1, in which the same spoligotype was also found in wild boar and red deer (*17*,*30*). No additional *M. bovis* isolates have been reported from this region, suggesting that after the initial introduction and spillover into local wild boar, the infection waned or persists at a low prevalence, in accordance with the predicted low risk for bovine TB in our model.

This observation suggests that relatively dense red deer populations are needed to maintain bovine TB at a high prevalence in wild ungulate populations. This finding also contrasts with the situation reported from south-central Spain, where wild boar is considered the main maintenance host, probably because intensive game management is rare in Portugal, with similarities with what was described for the Atlantic bioregion of Spain (*14*,*17*). Further studies involving spatially structured estimation of the abundance of wild ungulates and bovine TB prevalence along the edge of the infected area are warranted to identify the relative roles of wild boar and red deer densities in maintenance of bovine TB in this multihost system.

Red deer density and its interaction with the intensity of game management were associated with bovine TB in wild boar in the spatial risk model. Red deer density is a better predictor of bovine TB in wild boar populations than wild boar density, further strengthening the role of red deer as a key reservoir of bovine TB in wildlife in Portugal. The historical dynamics of wild boar populations are associated with contemporary distribution of bovine TB. Starting in the mid-20th century, wild boar populations expanded from historical refuges, and bovine TB in wildlife also seems to be increasing but at a much slower pace. One possible explanation for this pattern is that as wild boar populations spread, densities at the front of the expansion wave were low (*34*). Thus, bovine TB could not be maintained even with the addition of infected animals from infected historical refuges. As expansion continued and wild boar densities increased (*34*), the range of *M. bovis* seems to be slowly increasing.

The incidence of bovine TB in livestock was included in the selected model and highlighted the link between domestic and wild epidemiologic cycles in wildlife, which was strongly suspected on the basis of molecular epidemiology data (*30*). Although the directionality of such a link could not be inferred in our study, spillover from wildlife was shown to partially explain incidence of bovine TB in cattle in south-central Spain (*35*), suggesting that bovine TB in livestock could be an indicator of local disease transmission in wildlife.

In conclusion, we report a spatial analysis of bovine TB in wildlife in Portugal that used wild boar as a sentinel species and assessed the relative performance of dried blood spots collected on PS cards as a new sampling tool for large-scale serologic surveys. We confirmed the strong spatial clustering of bovine TB in wildlife and identified risk factors related to red deer density, intensity of wild ungulate management, historical population dynamics of wild boar, and incidence of bovine TB in cattle. The risk maps developed provide new tools for the targeted control of bovine TB in wild ungulate populations and identify areas at high risk for spread of disease.
